# Effect of Soft Tissue Thickness on Crestal Bone Levels and Implant Stability With Platform-Switched Abutments: A Prospective Study

**DOI:** 10.7759/cureus.88994

**Published:** 2025-07-29

**Authors:** Morupuri Sunitha, Thota K Mohan, Gottumukkala Vineela, Naga Sai Aneesha, Navya K Chowdary, Kura Srikanth

**Affiliations:** 1 Department of Prosthodontics, Sibar Institute of Dental Sciences, Guntur, IND; 2 Department of Periodontics, Meghna Institute of Dental Sciences, Nizamabad, IND

**Keywords:** bone loss, dental implant, mucosa, platform switching, soft tissue

## Abstract

Introduction: Soft tissue thickness may play a role in the success of dental implants by influencing factors such as bone health and implant stability. This study aimed to evaluate the effect of mucosal thickness on crestal bone levels and implant stability in patients with platform-switched abutments. The objectives were to measure bleeding on probing (BOP), implant stability, and changes in crestal bone levels in thin and thick mucosal environments over six months post-prosthetic loading.

Materials and methods: This prospective study included 20 partially edentulous patients divided into two groups: Group 1 (thin mucosa, <1 mm) and Group 2 (thick mucosa, 1-2 mm). Mucosal thickness was measured using a Mani K-file no. 20 (Mani, Utsunomiya, Japan). Two-piece titanium implants (3.7 mm diameter, 10 mm length, Alpha Bio, Petah Tikva, Israel) were placed using a surgical stent and sequential drilling with a physiodispenser (W&H, Bürmoos, Austria). Implant stability was assessed by resonance frequency analysis using an Osstell device (Osstell AB, Göteborg, Sweden). Crestal bone levels were measured using intraoral periapical radiographs with an X-ray grid. Platform-switched (PS) abutments were placed at three months, followed by screw-retained metal ceramic crowns. Clinical parameters (BOP, mucosal thickness, implant stability, and crestal bone levels) were evaluated at baseline, three months during the second surgery, and six months post-prosthetic loading. Data were analyzed using parametric and nonparametric tests with significance set at p < 0.05.

Results: The thick mucosa group exhibited significantly less crestal bone loss and higher implant stability at six months than the thin mucosa group (p < 0.05). BOP was absent in both groups of patients. Mucosal thickness remained stable, with significant differences between groups at all time points (p = 0.001). Implant stability improved over time in both groups (p = 0.001).

Conclusion: PS abutments promoted favorable peri-implant outcomes, with thicker mucosa associated with reduced crestal bone loss and enhanced stability, highlighting the role of mucosal thickness in optimizing implant success.

## Introduction

The rehabilitation of missing teeth using dental implants has become the cornerstone of modern prosthodontics, offering a sophisticated alternative to traditional tooth replacement methods [1]. Unlike conventional approaches that may involve adjacent healthy teeth, dental implants serve as artificial roots, providing robust mechanical support for both fixed and removable prostheses without compromising natural dentition [1]. Hailed as the "third dentition," implants have gained widespread acceptance owing to their osseointegration properties and biological compatibility, which underpins their long-term success [2]. The foundational concepts of osseointegration introduced by Brånemark [2] and functional ankylosis proposed by Schroeder et al. [3] have propelled implant dentistry over the past two decades, establishing it as a reliable treatment for fully or partially edentulous patients.

Despite these advancements, the prevention of crestal bone loss (CBL), particularly following abutment placement, remains a significant challenge in implantology [4]. Albrektsson et al. [5] noted that a successful implant may lose approximately 1.5 mm of crestal bone in the first year postoperatively, with an additional marginal loss of less than 0.2 mm annually thereafter. Several factors contribute to this phenomenon, including traumatic surgical procedures, stress concentration from occlusal loading at the implant-abutment junction (IAJ), microgaps fostering bacterial colonization, establishment of biological width, peri-implant inflammatory infiltrates, micromovements of prosthetic components, and repetitive screwing and unscrewing [6,7]. These factors can disrupt the implant-tissue interface, initiating bone loss in the crestal region and compromising both esthetic outcomes and implant stability.

To address CBL, innovative approaches such as non-submerged implants, scalloped designs, microthreaded implant necks, progressive loading, and immediate implant placement have been explored [8]. Among these, platform switching has garnered significant attention [9]. Platform switching involves using prosthetic abutments with a diameter smaller than the implant fixture, shifting the IAJ and associated inflammatory response medially away from the crestal bone [10]. This technique reduces marginal bone loss, optimizes esthetics, enhances bone-to-implant contact, and improves primary stability [9,10]. The widespread adoption of platform switching by major implant manufacturers underscores its clinical relevance.

The interplay between platform switching and soft tissue thickness is another critical factor influencing crestal bone preservation. Farronato et al. [11] concluded that platform switching appears to inhibit the recession of peri-implant soft tissues over an extended period when compared with implants devoid of any dimensional alteration of the abutment. Thicker soft tissues around edentulous sites have been shown to facilitate crestal bone preservation, complementing the benefits of platform-switched (PS) abutments. In an examination of peri-implant soft tissue associated with implants, Fu et al. [12] conducted a study examining the thickness of soft tissue surrounding implant-supported restorations and the influence of both thin and thick soft tissue biotypes. The results substantiated the significance of soft tissue biotype as a critical factor in the esthetic success of implant restorations, enhancing immediate implant outcomes and mitigating the risk of future mucosal recession [12]. However, the precise impact of varying soft tissue thicknesses on crestal bone levels in the context of platform-switched (PS) abutments remains unclear, necessitating further investigation.

The aim of this study was to evaluate the effect of mucosal thickness on crestal bone levels and implant stability in the presence of PS abutments. The objectives were to assess the presence or absence of bleeding on probing (BOP) in thin and thick mucosa with PS abutments, evaluate the stability of implants in these different mucosal environments, and analyze changes in crestal bone levels associated with PS abutments in thin and thick mucosa. These parameters were investigated to understand the role of soft tissue thickness in optimizing implant outcomes and minimizing CBL.

## Materials and methods

Study design and setting

This prospective study was conducted at the Department of Prosthodontics, Sibar Institute of Dental Sciences, Guntur, India. All patients provided written informed consent after a detailed explanation of the study procedures, risks, and benefits. Ethical approval was obtained from the Institutional Ethical Committee (IEC) of the Sibar Institute of Dental Sciences, Guntur, India (Pr.75/IEC/SIBAR/2022), ensuring compliance with ethical standards for clinical research. This study was conducted in accordance with the principles of the Declaration of Helsinki.

Sample size estimation

The sample size was calculated using G*Power software (version 3.1.9.2, Heinrich-Heine-Universität Düsseldorf, Düsseldorf, Germany). Based on a large effect size of 0.57 obtained from a previous study [13] analyzing crestal bone levels in PS abutments, the analysis determined that a minimum of 20 patients would be sufficient. This estimation was performed using a repeated-measures, a priori approach for three measurement time points (baseline, three months, and six months) with a power of 80% and an alpha error rate of 5%. Statistical significance was defined as p < 0.05.

Eligibility

Twenty systemically healthy patients aged 20-60 years with partial edentulism in mandibular first molar region were included. The eligibility criteria required sufficient bone height and width, D2 bone density as assessed by cone beam computed tomography (CBCT), and a minimum of 1 mm buccolingual keratinized gingiva. The exclusion criteria were chronic systemic diseases, recent head and neck radiation therapy, smoking or alcohol dependency, and pregnancy or lactation. Thorough medical and dental histories were recorded, and complete blood tests were performed to confirm eligibility and rule out contraindications for the implant surgery.

Pre-surgical phase

Twenty partially edentulous sites were selected and divided into two groups based on mucosal thickness: Group 1 (thin mucosa, <1 mm) and Group 2 (thick mucosa, 1-2 mm). Mucosal thickness was measured using a Mani K-file no. 20 (Mani, Utsunomiya, Japan) with a rubber stopper at four points (buccal mesial, buccal distal, lingual mesial, and lingual distal) on the attached gingiva between the mucogingival junction and an imaginary line from the cementoenamel junction (CEJ) of the adjacent teeth. The average of these measurements was used to categorize the patients into Group 1 (n = 10) (<1 mm, thin mucosa) or Group 2 (n = 10) (1-2 mm, thick mucosa). Diagnostic impressions were made using Algitex irreversible hydrocolloid impression material (DPI, Uttarakhand, India), and type III dental stone (Kalabhai, Mumbai, India) was poured to obtain diagnostic casts. A preoperative CBCT scan was performed to assess the bone height and width for implant placement. Prophylactic antibiotics were prescribed one day prior to surgery, such as amoxicillin (500 mg or 1 g orally, typically 1 h before surgery, or 2 g as a single dose), clindamycin (600 mg orally), or azithromycin (500 mg orally) for penicillin-allergic patients.

Surgical stent fabrication

Diagnostic casts were used to determine the implant position. A reference line (line A) was drawn along the center of the edentulous ridge crest, extending from the central fossa of adjacent teeth. A second line (line B) was drawn perpendicular to line A at the center of the edentulous space, extending buccolingually. The intersection of lines A and B indicates the implant position. An acrylic tooth (Primadent, Super Dental Products, Delhi, India) was placed in the edentulous region, and a wax-up was performed using modelling wax (Maarc, Mumbai, India). A separating medium was applied, and a surgical stent was fabricated using autopolymerizing clear acrylic resin (DPI, Uttarakhand, India). Reference lines were transferred to the stent, and a 2 mm hole was drilled at the intersection using a No. 6 round tungsten carbide bur (Marathon, Tokyo, Japan) to guide the pilot drill.

Surgical phase

Patients rinsed their mouths with 0.1% chlorhexidine mouthwash (ICPA Health Products, Ankleshwar, India) for two minutes, and the perioral mucosa was disinfected with povidone-iodine solution (PSK Pharma Pvt. Ltd., Karnataka, India). The mucosal thickness was re-evaluated, and BOP was assessed. Local anesthesia (Lignox 2% with 1:80,000 adrenaline; Indoco Remedies Ltd., Mumbai, India) was administered. A crestal incision with relieving incisions was made, and a full-thickness mucoperiosteal flap was created using a periosteal elevator (GDC, Punjab, India). The surgical stent guided a 2 mm pilot drill to mark the implant site. Sequential drilling (2.8 mm, 3.2 mm) was performed under copious saline irrigation (Abaris Healthcare Pvt. Ltd., Raipur, India) using a physio-dispenser (W&H, Bürmoos, Austria) at 400-800 rpm. Drilling depth was controlled using depth-marked drills or a depth gauge to ensure the osteotomy reached the predetermined 10 mm depth required for the implant length. A parallel pin ensured the correct angulation. Two-piece titanium implants (3.7 mm diameter, 10 mm length, Alpha Bio, Petah Tikva, Israel) were threaded into the osteotomy site at 25 rpm with a maximum torque of 50 Newton-centimeters (Ncm) until they were flush with the alveolar crest. Implant stability was measured using resonance frequency analysis (RFA) with an Osstell ISQ-RFA device (Osstell AB, Goteborg, Sweden), yielding implant stability quotient (ISQ) values (0-100). Nonabsorbable silk sutures (Johnson & Johnson Pvt. Ltd., Aurangabad, India) were placed. Intraoral periapical radiographs (IOPA) were obtained using a paralleling technique with a customized film holder and an X-ray grid to ensure consistent angulation at each time point (baseline, three months, and six months). The film holder was aligned with the long axis of the implant, and a standardized X-ray beam angulation was maintained using a positioning device to replicate the same orientation. Crestal bone height was measured as the distance from the implant shoulder to the alveolar crest at mesial and distal sites, with averages computed for analysis. Postoperative medications and chlorhexidine mouthwash were prescribed for four weeks, with sutures removed after one week.

Second surgical phase and prosthesis placement

At three months, soft and hard tissue parameters (mucosal thickness, BOP, implant stability, and crestal bone levels) were evaluated. The cover screw was replaced with a healing abutment for one week to allow the gingival contouring. PS abutments (Alpha Bio, Petah Tikva, Israel) with a diameter smaller than the 3.7 mm implant platform were used to create a horizontal offset at the implant-abutment junction (IAJ). The abutments were designed to shift the IAJ medially, reducing stress on the crestal bone and minimizing microgap-related bacterial infiltration. The PS abutments were made of titanium, compatible with the two-piece titanium implants, and were torqued to manufacturer-recommended specifications (typically 25-30 Ncm). The abutments were placed at three months post-implantation, following gingival contouring with a healing abutment, to support screw-retained metal-ceramic crowns.

The final impression was made using a closed-tray impression technique with putty and light-body elastomeric impression material (polyvinyl siloxane, DPI, Uttarakhand, India) in a stock tray. The putty was used to capture the overall arch anatomy, while the light-body material was applied around the transfer copings to ensure accurate reproduction of the implant position and surrounding soft tissue contours. The impression was taken after placing PS abutments to facilitate the fabrication of screw-retained metal-ceramic crowns. Transfer copings and implant analogs were used to pour a cast with a type IV die stone (Neelkanth Health Care Pvt. Ltd., Jodhpur, India). Screw-retained metal-ceramic crowns were fabricated with a screw access hole sealed with glass ionomer cement.

Follow-up and outcome assessment

**Figure 1:**
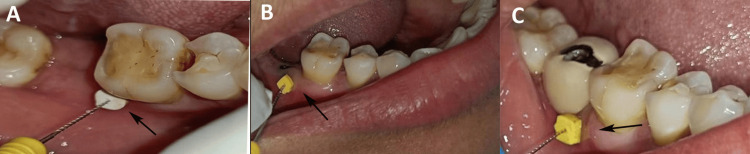
Mucosal thickness measured with Mani K-file no. 20 (black arrow) at baseline (A), three months at the second surgical phase (B), and six months post-prosthetic loading (C). Original images of a patient from the study, used with patient's consent.

Clinical parameters were assessed at baseline, three months at the second surgical phase, and six months post-prosthetic loading. Mucosal thickness was measured using Mani K-file no. 20 (MANI, Utsunomiya, Japan) and digital caliper (Figures 1A-1C).

**Figure 2:**
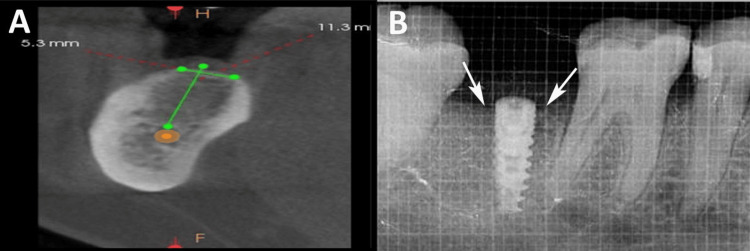
Measurement of alveolar bone levels in millimeters at preoperative phase (A) and assessment of crestal bone levels using grid method at three months on intraoral periapical radiographs (white arrows) (B). Original images of a patient from the study, used with patient's consent. Measurement unit of grid: 1 grid = 1 mm.

BOP was evaluated using a pressure-sensitive probe (GDC, Punjab, India) with 0.25 N/cm force, scored per the modified sulcular bleeding index as: 0 (no bleeding), 1 (pinpoint bleeding), 2 (thin linear rim), and 3 (profuse bleeding) [14]. Implant stability was measured by RFA using an Osstell device. CBL was assessed using IOPA radiographs with a grid (Figures 2A, 2B), measuring the distance from the implant shoulder to the alveolar crest at the mesial and distal sites, with averages computed.

Calibration and reliability

To ensure measurement consistency, a single trained examiner performed all clinical assessments. The examiner was calibrated by measuring mucosal thickness and BOP in five non-study patients, and intra-examiner reliability was assessed using the intraclass correlation coefficient (ICC > 0.9). Radiographic measurements were standardized using a grid and paralleling technique, with two independent evaluators analyzing IOPA images to confirm inter-examiner reliability (ICC > 0.85).

Statistical analysis

Data were analyzed using the Statistical Package for the Social Sciences (SPSS) version 20 software (IBM Corp., Armonk, NY, USA). The data were assessed for normality using the Shapiro-Wilk test. The crestal bone levels were found to deviate significantly from a normal distribution (p < 0.05), indicating a nonparametric distribution. Accordingly, nonparametric statistical tests were used for analysis of the crestal bone levels. Continuous variables, mucosal thickness, and implant stability were analyzed using the independent t test for intergroup comparisons. Additionally, intragroup differences across multiple time points were assessed using the repeated analysis of variance (ANOVA) or Friedman test, followed by post hoc analysis with Bonferroni or Conover tests for multiple comparisons. Differences were considered statistically significant at p < 0.05.

## Results

The demographic details of the study population are given in Table 1. The study included 20 patients, with 12 (60%) males and eight (40%) females. The mean age was 36.34 ± 6.65 years for males and 38.34 ± 5.65 years for females, showing a slightly higher age in females but with overlapping standard deviations, suggesting no major age disparity between genders.

**Table 1:** Demographic details of study sample. Age is presented as mean and standard deviation (SD). Number of patients in each gender are presented as frequency (N) and percentage (%).

Parameter	Category	Male	Female
Gender	N (%)	12 (60%)	8 (40%)
Age (in years)	Mean ± SD	36.34 ± 6.65	38.34 ± 5.65

At baseline, mucosal thickness differed significantly between the thin and thick groups, whereas stability and crestal bone levels were comparable. At three months, stability increased in both groups, with the thick group showing better stability. CBL was observed in both groups, with less CBL in the thick group at six months post-prosthetic loading. At six months, stability further improved in both the groups with a greater improvement in thick mucosa. BOP was absent in all the cases. The thick group demonstrated better stability and less CBL over time, suggesting that mucosal thickness may influence peri-implant outcomes (Table 2).

**Table 2:** Descriptive analysis of outcome variables in study groups at multiple time intervals. ISQ: implant stability quotient. Data are presented as mean, standard deviation (SD) and median. N denotes number of patients in each group.

Time	Group based on mucosal thickness	N (%)	Mucosal thickness (mm)		Stability (ISQ)		Crestal bone levels (mm)		Bleeding on probing	
			Mean ± SD	Median	Mean ± SD	Median	Mean ± SD	Median	Present (N)	Absent (N)
Baseline	Thin	10 (50%)	0.83 ± 0.09	0.80	54.30 ± 4.44	54.5	0.00 ± 0.00	0	0	10
	Thick	10 (50%)	1.82 ± 0.12	1.70	56.80 ± 4.05	56	0.00 ± 0.00	0	0	10
3 months at second stage surgery	Thin	10 (50%)	0.86 ± 0.08	0.75	61.50 ± 4.86	62	1.00 ± 0.00	1	0	10
	Thick	10 (50%)	1.85 ± 0.11	1.75	63.40 ± 4.58	62.5	0.70 ± 0.48	1	0	10
6 months post-prosthetic loading	Thin	10 (50%)	0.86 ± 0.08	0.75	70.60 ± 7.73	71	1.30 ± 0.48	1	0	10
	Thick	10 (50%)	1.85 ± 0.11	1.75	77.40 ± 7.95	80	0.50 ± 0.52	0.5	0	10

Intergroup comparisons revealed significant differences in mucosal thickness at all time points (p = 0.001), with the thicker group consistently showing higher mean value. Stability showed no significant difference at baseline (p = 0.205) and three months (p = 0.380) but became significant at six months (p = 0.048), favoring the thick group. The crestal bone levels remained comparable at three months; however, they differed significantly at six months (p = 0.006), with a greater CBL in the thin group. These findings suggest that while mucosal thickness differences persist, their impact on stability and bone loss becomes significant only after a longer follow-up period (Table 3).

**Table 3:** Intergroup comparison of outcome variables at multiple time intervals. *p < 0.05 denotes statistical significance. ISQ: implant stability quotient. Independent t test applied for mucosal thickness and stability. Mann-Whitney U test applied for crestal bone levels.

Time	Group based on mucosal thickness	Mucosal thickness (in mm)			Stability (ISQ)			Crestal bone levels (in mm)		
		Mean ± SD	t stat	p-value	Mean ± SD	t stat	p-value	Mean rank	U stat	p-value
Baseline	Thin	0.83 ± 0.09	19.6	0.001*	54.30 ± 4.48	-1.3	0.205	10.50	0.0	0.999
	Thick	1.82 ± 0.12			56.80 ± 4.05			10.50		
3 months at second stage surgery	Thin	0.86 ± 0.08	21.5	0.001*	61.50 ± 4.85	-0.9	0.38	12.00	42.1	0.281
	Thick	1.85 ± 0.11			63.40 ± 4.57			9.00		
6 months post-prosthetic loading	Thin	0.86 ± 0.08	21.5	0.001*	70.60 ± 7.73	-1.9	0.048*	13.75	82.5	0.006*
	Thick	1.85 ± 0.11			77.40 ± 7.94			7.20		

Intragroup comparison of thin and thick mucosal phenotypes across three time points was performed using repeated measures ANOVA for mucosal thickness/implant stability and the Friedman test for crestal bone levels. For thin mucosa, thickness increased significantly from baseline (0.83 ± 0.09 mm) to three months (10.86 ± 0.08 mm, p = 0.001) and was maintained until six months (0.86 ± 0.08 mm). Stability (ISQ) progressively improved (54.30 ± 4.48 to 61.50 ± 4.85, p = 0.001), whereas crestal bone levels deteriorated (p = 0.001). Thick mucosa showed inverse thickness changes ( p = 0.001), with greater ISQ gains (63.40 ± 4.57 to 77.40 ± 7.94, p = 0.001) and stable bone levels (p = 0.004). Thick mucosa demonstrated superior stability (higher ISQ) and bone preservation despite cyclical thickness changes, whereas thin mucosa showed persistent bone loss despite stability improvements. The mucosal phenotype significantly influenced peri-implant healing, with thick mucosa offering better long-term outcomes (Table 4).

**Table 4:** Intragroup comparison at multiple time intervals for outcome variables. *p < 0.05 denotes statistical significance. ISQ: implant stability quotient. Repeated analysis of variance (ANOVA) test was applied for mucosal thickness and stability. Friedman test was applied for crestal bone levels.

Group based on mucosal thickness	Time	Mucosal thickness (in mm)			Stability (ISQ)			Crestal bone levels (in mm)		
		Mean ± SD	F stats	p-value	Mean ± SD	F stats	p-value	Mean rank	Chi stats	p-value
Thin	Baseline	0.83 ± 0.09	45.76	0.001*	54.30 ± 4.48	41.86	0.001*	0.0	18.72	0.001*
	3 months at second stage surgery	0.86 ± 0.08			56.80 ± 4.05			11.9		
	6 months post-prosthetic loading	0.86 ± 0.08			61.50 ± 4.85			12		
Thick	Baseline	1.82 ± 0.12	45.23	0.001*	63.40 ± 4.57	48.7	0.001*	0	11.14	0.004*
	3 months at second stage surgery	1.85 ± 0.11			70.60 ± 7.73			13.75		
	6 months post-prosthetic loading	1.85 ± 0.11			77.40 ± 7.94			7.25		

Mucosal thickness showed significant changes from baseline to three and six months (p = 0.001 for both the groups) but no difference between three and six months (p = 0.999). Implant stability improved significantly from baseline to three months and further at six months, with continued improvement between three and six months. CBL was significant from baseline to six months in both groups, although the thin mucosa group exhibited greater CBL than the thick mucosa group. Thick mucosa correlated with better stability and reduced bone loss over time, whereas thin phenotypes were prone to progressive bone resorption. Early stability gains occur within three months; however, bone preservation requires phenotype-specific interventions (Table 5).

**Table 5:** Post hoc analysis for outcome variables. *p < 0.05 denotes statistical significance: ISQ: implant stability quotient. Bonferroni post hoc applied for mucosal thickness and stability. Conover's post hoc test applied for crestal bone levels.

Multiple time interval	Mucosal thickness in mm				Stability (ISQ)				Crestal bone levels in mm			
	Thin		Thick		Thin		Thick		Thin		Thick	
	t stat	p-value	t stat	p-value	t stat	p-value	t stat	p-value	t stat	p-value	t stat	p-value
Baseline vs. 3 months	-6.7	0.001*	-6.7	0.001*	-5.2	0.002*	-5.8	0.001*	12.4	0.001*	4.6	0.001*
Baseline vs. 6 months	-6.7	0.001*	-6.7	0.001*	-6.7	0.001*	-7.3	0.001*	15.2	0.001*	3.3	0.004*
3 months vs. 6 months	0	0.999	0	0.999	-6.8	0.001*	-6.6	0.001*	2.7	0.012*	1.3	0.203

## Discussion

The study found significantly less CBL in the thick mucosa group than in the thin mucosa group at six months (p = 0.01). This suggests that a thicker mucosa may offer a protective effect against crestal bone resorption. The biological rationale for this finding lies in the enhanced soft-tissue barrier provided by the thicker mucosa, which may better shield the underlying bone from microbial invasion and stress. Thicker mucosa also supports improved blood supply and tissue stability, potentially reducing bone remodeling around implants [15].

Several studies have corroborated these findings. Linkevicius et al. [16] demonstrated that implants placed in thin mucosa exhibited greater CBL than those placed in thicker mucosa, which is attributed to the limited soft tissue volume that fails to establish an adequate biological width. Similar findings were reported in a systematic review by Suárez-López Del Amo et al. [17], who reported that a thicker peri-implant mucosa was associated with reduced CBL, emphasizing the role of soft tissue in maintaining the bone stability. These studies align with the present findings, suggesting that a thicker mucosa creates a more robust peri-implant seal, minimizing CBL.

In contrast, some studies have reported a minimal influence of mucosal thickness on the CBL. Canullo et al. [18] found no significant difference in CBL between the thin and thick mucosa groups when using PS implants, suggesting that PS itself may mitigate bone loss, regardless of soft tissue thickness. This discrepancy could be attributed to differences in implant design, surgical protocols, or follow-up duration. In the present study, the standardized use of PS abutments and six-month follow-up may have amplified the influence of mucosal thickness, as bone remodeling may be more pronounced in the early post-implantation period.

The significant CBL difference observed in this study may be due to the biomechanical advantages of a thicker mucosa, which provides a cushioning effect against occlusal forces and reduces stress concentration at the bone-implant interface [15]. Additionally, the thin mucosa group may have undergone a more pronounced biological width re-establishment, leading to increased bone resorption [19]. The use of PS abutments likely minimized CBL in both groups compared to non-PS designs, but the protective effect was more evident in the thick mucosa group owing to its enhanced soft-tissue barrier.

Implant stability improved significantly in both groups over six months, with the thick mucosa group showing superior stability at six months. This suggests that a thicker mucosa may enhance osseointegration by providing a stable soft tissue environment that supports bone-implant integration. The increased stability in the thick mucosa group may be linked to reduced micromovement at the implant site, facilitated by a more robust soft-tissue framework. A previous review article reported that thicker soft tissue is associated with higher ISQ values, likely owing to reduced micro-motion and improved tissue adaptation [20]. This suggests that soft tissue thickness plays a critical role in stabilizing implants during the healing phase.

Conversely, some studies have indicated that implant stability is primarily influenced by bone quality rather than by soft tissue thickness. Albrektsson et al. [21] and Merheb et al. [22] emphasized bone density as the primary determinant of stability, with soft-tissue thickness playing a secondary role. The lack of significant stability differences at baseline and three months in the present study partially aligns with this view, as initial stability may depend more on bone characteristics than on mucosal thickness. The significant difference in stability at six months in this study may reflect the cumulative effect of soft-tissue support on osseointegration. Thicker mucosa likely reduces peri-implant inflammation and micromovement and enhances bone-implant contact over time [17].

The absence of BOP in both groups suggests excellent peri-implant health, indicating that PS abutments may contribute to reduced inflammation regardless of mucosal thickness. The mechanism by which PS abutments contribute to reduced bleeding on probing involves an inward shift of the implant-abutment interface, which minimizes the microgap and bacterial infiltration at the crestal bone level [10]. By positioning the abutment diameter smaller than the implant platform, the platform switching creates a horizontal offset that repositions the inflammatory cell infiltrate away from the bone, thereby reducing peri-implant inflammation [23]. This configuration enhances the soft-tissue seal around the implant, limiting microbial penetration and subsequent tissue irritation [11]. Additionally, PS abutments promote more stable mucosal attachment and decrease mechanical trauma during probing [9,23]. In the present study, the absence of bleeding on probing in both the thin and thick mucosa groups underscored the efficacy of platform switching in maintaining peri-implant health in the long term. [8]. In contrast, Romanos and Javed [24] conducted a systematic review to assess the role of platform switching in CBL and concluded that seven studies reported no significant difference in CBL between PS and non-PS abutments.

The consistent absence of BOP in our study likely reflects the efficacy of PS abutments in reducing microleakage and the rigorous postoperative care protocol, including chlorhexidine rinsing. The use of a pressure-sensitive probe ensured standardized assessment and minimized false positives. The lack of bleeding on probing in both groups suggests that PS abutments are effective in maintaining peri-implant health, regardless of mucosal thickness.

Intragroup analysis revealed significant improvements in implant stability over time in both groups, with the thin mucosa group showing significant changes in CBL at three and six months postoperatively. The thick mucosa group exhibited borderline CBL changes, suggesting greater bone-level stability. Post hoc analysis confirmed that the CBL changes in the thin mucosa group were more pronounced, highlighting the vulnerability of thin biotypes to bone resorption. Berglundh et al. [25] reported that thin mucosa is associated with increased biological width formation, leading to greater CBL. This finding aligns with the significant CBL changes observed in the thin mucosa group in the present study. In contrast, Annibali et al. [26] found no significant CBL differences with PS abutments, suggesting that early bone remodeling may not persist over the long term. The pronounced CBL changes in the thin mucosa group may reflect the need for greater bone remodeling to establish biological width, as the thin mucosa provides less soft-tissue volume to accommodate this process. The borderline CBL changes in the thick mucosa group suggest a more stable peri-implant environment, potentially due to enhanced soft tissue support.

Clinical implications

The findings of this study highlight the significant role of soft tissue thickness in optimizing peri-implant outcomes when using PS abutments. Clinicians should prioritize preoperative assessment of mucosal thickness to predict CBL and implant stability. Patients with thin mucosa may benefit from soft tissue augmentation to mitigate higher CBL. The consistent absence of BOP in both groups might support the use of PS abutments to enhance peri-implant health by reducing inflammation and microbial infiltration. Regular monitoring at three and six months is recommended to track stability and CBL, particularly in thin biotypes where bone resorption is more pronounced. These insights suggest that tailoring implant protocols based on mucosal thickness can improve long-term success rates, with PS abutments being particularly effective in minimizing CBL and maintaining soft-tissue health.

Limitations

However, this study has several limitations. A sample size of 20 patients, though statistically sufficient, restricts the generalizability of the findings, and larger cohorts could provide more robust evidence. The six-month post-prosthetic loading follow-up period may not fully capture long-term CBL trends, as bone remodeling may continue beyond this timeframe. Reliance on a single trained examiner for clinical assessments, despite high intra-examiner reliability, introduces potential bias, and multiple examiners could enhance measurement consistency. The nonparametric distribution of data necessitates specific statistical tests, which may limit the robustness of the comparisons. Additionally, the study focused solely on PS abutments and did not compare them with non-PS designs, thus limiting insights into their relative efficacy. Future studies with larger sample sizes, longer follow-up periods, and comparative designs could address these limitations to further validate the findings.

## Conclusions

This study demonstrated that thicker mucosa (2 mm) was associated with significantly reduced crestal bone loss and enhanced implant stability compared to thinner mucosa (1 mm) when using PS abutments. The absence of bleeding on probing in both groups highlighted the effectiveness of PS abutments in maintaining peri-implant soft tissue health. These findings emphasize the critical role of mucosal thickness in optimizing peri-implant outcomes, with thicker mucosa providing a protective effect against bone resorption and improving long-term implant success.
